# Are Opioid Receptor Antagonists Effective at Treating Antipsychotic-Induced Weight Gain? A Systematic Review and Meta-Analysis

**DOI:** 10.1192/bjo.2024.265

**Published:** 2024-08-01

**Authors:** Kenn Cheng Keat Lee, Matthew Twohig, Nguemo Pauline Idoko, Benjamin David Williams

**Affiliations:** 1 Pennine Care NHS Foundation Trust, Bury, United Kingdom; 2 Pennine Care NHS Foundation Trust, Ashton-Under-Lyne, United Kingdom; 3 Greater Manchester Mental Health NHS Foundation Trust, Manchester, United Kingdom

## Abstract

**Aims:**

Introduction: Second-generation antipsychotics are widely used in psychiatry but are associated with weight gain. Obesity is more prevalent in mental illness and may contribute to the mortality gap. Non-pharmacological management of antipsychotic-induced weight gain (AIWG) has limited success whilst pharmacological treatment typically involves antidiabetic medications that psychiatrists have less experience with. Recent developments in the field have shown promise with using centrally-acting opioid receptor antagonists (CORAs) at treating AIWG.

Objective: Review and synthesise the available RCT evidence on the efficacy of CORAs at treating AIWG.

**Methods:**

Methodology: Four databases (Medline, Embase, PsycINFO, Cochrane) were searched, from database inception to present, for RCTs using CORAs (naloxone, naltrexone, samidorphan) to reduce AIWG. Our primary outcome sought was weight change in kilograms, with secondary outcomes of change in percentage of body weight, waist circumference and 7% or 10% weight change thresholds. We used random-effects meta-analysis due to study heterogeneity.

**Results:**

A total of 450 articles were found (319 post-deduplication), of which seven met criteria (samidorphan = 4, naltrexone = 3, naloxone = 0) including *n* = 1,416 patients. On meta-analysis, change in body weight (kg) for CORAs as a class was statistically significant (RE = 1.37 kg; 95% CI: 0.51, 2.24). However, change in BMI was not statistically significant (RE = 0.61kg/m^2^; 95% CI: −0.56, 1.78). Remaining analysis was only available for samidorphan, which showed statistically significant improvement in change in body weight (%) (RE = 1.81%; 95% CI: 1.07, 2.55), absolute risk of weight gain ≥7% (RE = 12.41%; 95% CI: 6.55, 18.27), absolute risk of weight gain ≥10% (RE = 10.83%; 95% CI: 5.46, 16.21), and change in waist circumference (RE = 1.50 cm; 95% CI: 0.32, 2.67).

**Conclusion:**

Evidence is strongest for samidorphan, though CORAs as a class remains poorly researched and the benefits are modest. Additionally, samidorphan is currently only available in the combination medication olanzapine-samidorphan and the literature reflects this. Further research is needed to examine its efficacy in AIWG from other antipsychotics.

